# Assessing the compliance of systematic review articles published in leading dermatology journals with the PRISMA statement guidelines: A systematic review protocol

**DOI:** 10.1016/j.isjp.2018.11.001

**Published:** 2018-11-15

**Authors:** Buket Gundogan, Alexander Fowler, Riaz Agha

**Affiliations:** a East and North Hertfordshire NHS Trust, Hertfordshire, UK; b Royal London Hospital, Barts Health NHS Trust, London, UK; c Department of Plastic Surgery, Royal Free Hospital NHS Foundation Trust, London, UK; d Chelsea and Westminster Hospital NHS Foundation Trust, London, UK

**Keywords:** Dermatology, Systematic reviews, Meta-analysis, Reporting quality

## Abstract

**Introduction:**

Systematic reviews and meta-analyses are methodologically rigorous studies that are said to form the reference standard for summarising evidence to guide health care. Reporting quality of reviews is of critical importance in order to judge the quality and risk of bias in a review to ensure sound healthcare decisions are made. This is particularly important in the field of dermatology due to the growing number of systematic reviews and their key role in informing healthcare decision within dermatology. A contemporary and comprehensive review of the compliance of dermatology systematic reviews and meta-analyses with the PRISMA checklist, in the highest impact factor dermatology journals, has not yet been assessed. To our knowledge, our review represents the most extensive study assessing reporting quality of systematic reviews and meta-analyses published within dermatology to date.

**Methods and analysis:**

Our protocol is reported in line with the Preferred reporting items for systematic review and meta-analysis protocols (PRISMA-P) 2015 guidelines. MEDLINE will be searched to look for systematic reviews and meta-analysis in selected years within the top four highest impact factor dermatology journals in 2017. Records and full texts will be screened independently by five researchers. Data will be extracted onto a standard data extraction database. A training session will take place to ensure accurate data extraction and scoring of studies with the PRISMA checklist. The data will be analysed and outcomes will be determined. Primary outcome will be the compliance of reviews with the PRISMA checklist.

## Highlights

• Reporting quality of systematic reviews is of critical importance.
• Reporting quality of reviews in leading dermatology journals is yet to be assessed.
• Hereby we present a protocol to assess reporting quality in dermatology reviews.

## 1 Introduction

Systematic reviews and meta-analyses are methodologically rigorous studies that are said to form the reference standard for creating evidence in health care [1]. In 1979, Archie Cochrane discussed the pressing need for the critical reviews of trials to be published, at a time when approximately 14 trials were published a day [2]. As of 2010 approximately 75 trials and 11 systematic reviews were published daily, with a plateau not yet reached [3]. These figures are likely to have increased.

With this immense growth in number of studies, it is critical now more than ever to ensure reporting quality and transparency are adhered to. Whilst reporting quality and study quality are not the same, a poorly reported study is of limited value since it is difficult to make a full and transparent judgment of its utility without all of the necessary information [4]. It is important to note that poor reporting quality may be related to poor underlying quality of the study, however this is not always necessarily the case. In fact, a well-conducted study may even be poorly reported and perhaps ‘masked’ by its reporting. It is well established that deviation from the reporting guidelines in systematic reviews and meta-analyses can lead to bias [5]. Such work has subsequently resulted in the development of the Preferred Reporting Items for Systematic Review and Meta-analysis (PRISMA) statement, a twenty-seven item checklist which ensures reporting transparency of a review [6].

Given the increase in systematic reviews within dermatology it is important, perhaps now more than ever, to ensure reviews are adequately reported [7]. Well-reported reviews will ensure clinicians and policy makers alike are able to make complete and transparent judgments to guide key healthcare decisions and ensure cost-effectiveness of treatments within dermatology.

## 2 Rationale

Systematic reviews and meta-analyses form a critical part of dermatological research since they form the reference standard for summarising evidence to guide decisions within clinical dermatology, whilst also minimising bias [8]. Therefore it is necessary that reviews published in this field are fully compliant with the PRISMA guidelines.

It has previously been reported that systematic reviews and meta-analyses within dermatology were less likely to evaluate publication bias [9]. Publication bias is one of the twenty-seven items in the PRISMA reporting guidelines. Other work published by the Cochrane Skin Group (CSG) assessed thirty-eight reviews on selected dermatological topics and demonstrated a higher methodological quality in CSG reviews compared to non-CSG reviews [10].

The compliance of dermatology systematic reviews and meta-analyses across all items of the checklist and in the highest impact factor journals is yet to be assessed. To our knowledge, our review will represent the most extensive study assessing reporting quality of systematic reviews and meta-analyses published within dermatology to date.

## 3 Objectives

### 3.1 Primary objective

This systematic review will assess the compliance of systematic reviews and meta-analyses in leading dermatology journal with the Preferred Reporting Items for Systematic Review and Meta-analysis (PRISMA) Statement.

### 3.2 Secondary objectives

To assess whether compliance of PRISMA guidelines improves over time and whether this correlates with mandatory enforcement of PRISMA reporting or appointment of a dedicated systematic review editor. To assess: the variety of sub-topics reviewed, if reviews are registered, if protocols exist and if there is a difference in PRISMA compliance between Cochrane vs non-Cochrane reviews, to rank in order of compliance the items of the checklist across all studies and to assess if certain items are consistently poorly reported. Lastly to assess if there is an assessment of publication bias across systematic reviews as well as within its primary studies.

### 3.3 Hypothesis

It is hypothesised that systematic reviews in leading dermatology journals are fully compliant with the PRISMA checklist. In addition to this, it is hypothesised that systematic reviews published more recently have higher compliance with the checklist.

## 4 Methods

This protocol is in line with the Preferred Reporting Items for Systematic Review and Meta-analysis Protocols (PRISMA-P) 2015 statement [1]. This review will be carried out in line with the Cochrane Handbook for Systematic Reviews and Interventions [17], and will be in line with the PRISMA guidelines [6], [11]. It has been registered *a priori* with the international prospective register of systematic reviews, Research Registry (review number: reviewregistry597) [12].

A search technique similar to a systematic review assessing the PRISMA compliance in craniofacial surgery reviews will be used, in order to increase comparability with previous similar work [13].

### 4.1 Eligibility criteria

Inclusion criteria will include: systematic reviews and meta-analyses within dermatology, reviews only published within the top four highest impact factor journals as of 2017, reviews published in the years 2016/17, 2011/12 and 2006/07 and English language studies. The selected dates were chosen since 2016/17 is the most contemporaneous two-year period and five-year periods proceeding these years were chosen to allow for comparators.

Exclusion criteria will include: articles that are not systematic reviews and meta-analyses, articles outside of the dates and journals previously mentioned, historical reviews, narrative literature reviews, grey literature or unpublished reviews.

### 4.2 Information sources

In order to locate the top four highest impact factor dermatology journals as of 2017, the Thomson Reuters InCites Journal Citation Reports was utilised (https://jcr.incites.thomsonreuters.com, Thomas Reuters, New York, US) [14]. The search term for the journals was the “dermatology” category and Journal Citation Report year selected was 2017. This search identified the top four journals as: JAMA Dermatology (JAMAD), Journal of the American Academy of Dermatology (JAAD), Journal of Investigative Dermatology (JID) and the British Journal of Dermatology (BJD).

MEDLINE PubMed will be searched for the reviews during the six years of 2016/17, 2011/12 and 2006/07. MEDLINE PubMed will be utilised since all four journals (JAMAD, JAAD, JID and BJD) are PubMed indexed.

### 4.3 Search strategy

The search strategy aims to collect all systematic reviews and meta-analyses within the selected journals during the years aforementioned. The strategy will be developed in line with an information search specialist. Search strategy will include the use of Boolean logical operators to improve sensitivity of the search. The search terms will be “systematic review” OR “meta-analysis” OR “meta-analyses” AND “JOURNAL NAME”.

An example full search strategy conducted on 1/8/18 includes: (“Br J Dermatol”[Journal] OR “british journal of dermatology”[All Fields]) AND “systematic review”[All Fields] AND (“2006/01/01”[PDAT]: “2006/12/31”[PDAT]).

### 4.4 Study records

There will be two stages to the screening and identification of study records. Firstly, five independent researchers will screen the titles and abstracts against inclusion and exclusion criteria. In the second stage of screening, full text will be assessed for inclusion and exclusion criteria. Any discrepancies between articles will be resolved by discussion or senior author decision (BG). Reasons for exclusion will be noted. Articles that have passed both stages of screening will be included for data extraction. Data will then be extracted onto a standard extraction database created on Google Forms (Alphabet Inc, California, USA). Duplicates will be removed before extraction.

A training session will take place before data extraction focussing on accurate marking of included studies against the PRISMA guidelines. This training session will be conducted by a senior researcher and involve “practice” marking of dermatology systematic reviews with the PRISMA checklist with any discrepancies within researchers being fed back to the team and discussed in training.

### 4.5 Data items

The following data items will be extracted from the articles: compliance of each article with the twenty-seven item PRISMA checklist, study authors, date of publication, journal name, dermatology sub-topic assessed by the review, country where review took place as assessed by first author, commercial or non-commercial funding, if a protocol exists, if it is a Cochrane review, if the review is registered. Overall outcome of the review will also be assessed with regard to whether the review showed a positive, equivocal or negative outcome with respect to the intervention being studied. Dermatology sub-topics will be divided using the Cochrane Skin Group titles categorised by the British Association of Dermatologists (BAD) diagnostic index [15]. Further two sub-topics added will be educational and meta-research for the purposes of this review.

### 4.6 Article scoring

Articles will be scored for compliance with the twenty-seven item PRISMA checklist. A score of one will be given for an article that meets all the criteria for a particular item, a score of zero for those that do not meet or partially meet the item requirements and not-applicable (N/A) if there are further concerns or if the particular item is not relevant to the article. For some studies, not all twenty-seven items of the checklist will be relevant in which case the maximum PRISMA score of an article will be calculated by subtracting the number of irrelevant items by twenty-seven. A compliance score expressed as a percentage will be calculated for each article.

### 4.7 Outcomes and prioritisation

Primary outcome will be compliance with the PRISMA checklist. Secondary outcomes assessed will be to assess whether compliance of PRISMA guidelines improves over time and whether this correlates with mandatory enforcement of PRISMA reporting or appointment of a dedicated systematic review editor. Other outcomes will be to describe sub-topics reviewed, to assess if reviews are registered, if protocols exist and if there is a difference in PRISMA compliance between Cochrane vs non-Cochrane reviews, to rank in order of compliance the items of the checklist across all studies and to assess if certain items are consistently poorly reported. Lastly to assess if there is an assessment of publication bias across systematic reviews as well as within its primary studies.

### 4.8 Assessing bias and meta-bias

This will be assessed as to whether a systematic review or meta-analysis is compliant with PRISMA checklist items pertaining to bias, which is as follows: assessment of risk of bias in individual studies, assessment of risk of bias across studies, presenting data on the risk of bias within studies, presenting data on the risk of bias across studies.

### 4.9 Data synthesis

Data will be analysed and assessed using Microsoft Excel (Microsoft, Redmond, WA, USA). Continuous variables will be presented by their mean and ranges and categorical variables will be presented as percentages. The PRISMA compliance will be expressed as a percentage for each study. A preliminary pilot search conducted on 1/8/18 has identified 233 reviews and meta-analyses for screening.

### 4.10 Sub-group analyses

The PRISMA score across all articles will be compared based on various factors such as: journal impact factor, year published, sub-topics, presence of a protocol, *a priori* registration status. There will also be analysis of the checklist items that are consistently most and least compliant with ranking. No meta-regression or sensitivity analyses will be planned.

### 4.11 Ethics and dissemination

The authors aim to disseminate the results as widely as possible. The systematic review will have published in a peer-reviewed journal and will be presented in a broad range of national and international conferences. We will also be carrying out this review in line with PRISMA-2015 statement. This review will be carried out in line with Cochrane Handbook for Systematic Reviews and Interventions and will be compliant with PRISMA guidelines.

## 5 Conclusion

By conducting this systematic review, we aim to clarify deficiencies in systematic review reporting, contribute to improvement of methodology and therefore transparency of reporting within dermatology systematic reviews and meta-analyses. As a result, we hope this will ultimately ensure good clinical practice and patient care.

## Ethical approval

Not applicable.

## Funding

The authors declare no commercial or other sources of funding for this review.

## Author contribution

BG and RA conceived idea for the study. BG drafted the manuscript. BG, RA and AJF authors critically revised the manuscript for intellectual content and approved the final version for publication. BG is guarantor for the review.

## Conflict of interest statement

None declared. No funding or sponsorship received for this study.

## Guarantor

BG is guarantor for the review.

## Research Registration Number

reviewregistry597.
